# Development of an AI Model for Predicting Methacholine Bronchial Provocation Test Results Using Spirometry

**DOI:** 10.3390/diagnostics15040449

**Published:** 2025-02-12

**Authors:** SangJee Park, Yehyeon Yi, Seon-Sook Han, Tae-Hoon Kim, So Jeong Kim, Young Soon Yoon, Suhyun Kim, Hyo Jin Lee, Yeonjeong Heo

**Affiliations:** 1 Biomedical Research Institute, Kangwon National University Hospital, Chuncheon 24289, Republic of Korea; hamusj627@gmail.com; 2 Department of Internal Medicine, Seoul Medical Center, Seoul 02053, Republic of Korea; kamikat@naver.com (Y.Y.); sammygg@seoulmc.or.kr (S.K.); 3 Department of Internal Medicine, Kangwon National University, Chuncheon 24341, Republic of Korea; ssunimd@naver.com (S.-S.H.); blessing0104@naver.com (T.-H.K.); 4 Division of Pulmonary, Allergy and Critical Care Medicine, Department of Internal Medicine, Hallym University Dongtan Sacred Heart Hospital, Hwaseong 18450, Republic of Korea; areumi0716@naver.com; 5 Division of Pulmonary and Critical Care Medicine, Department of Internal Medicine, Dongguk University Ilsan Hospital, Goyang 10326, Republic of Korea; ysyoonmd@gmail.com; 6 Internal Medicine, Seoul National University Seoul Metropolitan Government Boramae Medical Center, Seoul 07061, Republic of Korea; x8uisol8z@naver.com

**Keywords:** methacholine bronchial provocation test, machine learning, asthma

## Abstract

**Background/Objectives**: The methacholine bronchial provocation test (MBPT) is a diagnostic test frequently used to evaluate airway hyper-reactivity. MBPT is essential for diagnosing asthma; however, it can be time-consuming and resource-intensive. This study aimed to develop an artificial intelligence (AI) model to predict the MBPT results using forced expiratory volume in one second (FEV_1_) and bronchodilator test measurements from spirometry. **Methods**: a dataset of spirometry measurements, including Pre- and Post-bronchodilator FEV_1_, was used to train and validate the model. **Results**: Among the evaluated models, the multilayer perceptron (MLP) achieved the highest area under the curve (AUC) of 0.701 (95% CI: 0.676–0.725), accuracy of 0.758, and an F1-score of 0.853. Logistic regression (LR) and a support vector machine (SVM) demonstrated comparable performance with AUC values of 0.688, while random forest (RF) and extreme gradient boost (XGBoost) achieved slightly lower AUC values of 0.669 and 0.672, respectively. Feature importance analysis of the MLP model identified key contributing features, including Pre-FEF_25–75_ (%), Pre-FVC (L), Post FEV_1_/FVC, Change-FEV_1_ (L), and Change-FEF_25–75_ (%), providing insight into the interpretability and clinical applicability of the model. **Conclusions**: These results highlight the potential of the model to utilize readily available spirometry data, particularly FEV_1_ and bronchodilator responses, to accurately predict MBPT results. Our findings suggest that AI-based prediction can improve asthma diagnostic workflows by minimizing the reliance on MBPT and enabling faster and more accessible assessments.

## 1. Introduction

In patients presenting with a chronic cough, cough-variant bronchial asthma should be regarded as a primary differential diagnosis [1,2]. Asthma is a leading cause of chronic coughs, accounting for 24% to 29% of cases in non-smokers, as reported in several prospective studies [3]. However, a definitive diagnosis requires a bronchial provocation test using methacholine or histamine [4,5]. Methacholine is a pharmacological agent that induces bronchial constriction and impairs respiratory function. Therefore, a bronchial provocation test involving the inhalation of aerosolized methacholine is commonly employed in clinical settings to diagnose airway hyperresponsiveness [6,7]. A decrease of 20% or more in forced expiratory volume in 1 s (FEV_1_) following methacholine inhalation is a widely accepted criterion for confirming bronchial hyperresponsiveness [7]. The methacholine bronchial provocation test (MBPT), a diagnostic method for asthma, measures airway hyperresponsiveness, a key indicator of asthma. PC20 refers to the concentration of methacholine that results in a 20% decrease in FEV_1_ from baseline and serves as an important diagnostic criterion. For PC20 < 1.0 mg/mL, the specificity and precision are approximately 100%, enabling a definitive asthma diagnosis. A PC20 value of between 8 and 16 mg/mL indicates airway hyperresponsiveness, while a PC20 ≥ 16 mg/mL makes it highly unlikely that the patient has active asthma [8]. Despite its reliability and high negative predictive value, MBPT usage is limited by cost, time requirements, and restrictions for patients undergoing treatment with steroids or antihistamines for bronchial asthma concurrently [9].

Otherwise, forced spirometry is a reliable diagnostic method for bronchial asthma [10,11]. The bronchodilator test evaluates changes in FEV_1_ following inhalation of a short-acting bronchodilator [12], such as salbutamol, a short-acting beta2-agonist (SABA). Forced spirometry is relatively less expensive, and can be performed regardless of the patient’s medication status. Additionally, this value has demonstrated test reproducibility independent of patient effort [13]. However, it is not recommended solely for asthma screening due to its low sensitivity [14].

The forced mid-expiratory flow between 25% and 75% (FEF_25–75_) of forced vital capacity (FVC), which is indicated while the spirometry is performed, is considered a more sensitive marker of small airway function than FEV_1_ [15,16]. The clinical utility of FEF_25–75_ remains less studied compared to FEV_1_, which is a well-established marker of airway obstruction. Nonetheless, some evidence suggests that FEF_25–75_ correlates well with airway hyperresponsiveness and holds clinical relevance in the sensitive diagnosis of asthma [17,18]. It has been suggested that impaired FEF_25–75_ may precede FEV_1_ impairment and serve as an early marker of bronchial obstruction [19]. Despite its potential clinical relevance, skepticism exists regarding the interpretation of FEF_25–75_ due to its critical dependence on FVC [20].

Given the variability in lung function and symptoms in asthma, previous studies have explored predictive models incorporating FEF_25–75_, FVC, FEV_1_, and MBPT outcomes [21,22,23]. However, these approaches face limitations, including reproducibility challenges, reliance on patient cooperation, and independence on treatment status. Recently, artificial intelligence (AI) approaches have been introduced for asthma diagnosis; however, clinical applications remain limited, and further studies are needed to generalize the findings [24,25]. This study aimed to develop an explainable AI model to predict MBPT results using a forced spirometry performed following bronchodilator administration, and its use as an adjunct for asthma diagnosis.

## 2. Materials and Methods

### 2.1. Study Population

We used a dataset collected from multiple centers in the Republic of Korea. The data used in this study were obtained with the approval of the Institutional Review Boards (IRB) of Kangwon National University Hospital (KNUH-2024-03-004), Seoul Medical Center (2024-04-003), Dongguk University Ilsan Hospital (2024-08-017-001), and Hallym University Dongtan Sacred Heart Hospital (HDT 2024-10-004). The criteria for data selection included patients aged 18 years or older who underwent both a spirometry and a MBPT after bronchodilator administration. At this time, data were collected for cases in which the interval between the two tests was within 2 months and spirometry was performed before MBPT.

### 2.2. Data Collection

We used 20 variables related to pulmonary function tests, including FEV_1_, FVC, and FEF_25–75_, to develop a model for predicting MBPT results. The MBPT results were categorized into a binary classification based on a threshold of 16 mg/mL: results less than 16 mg/mL were one category, while results of 16 mg/mL or greater were the other category. Among the data, 3498 results were 16 mg/mL or greater, and 862 results were less than 16 mg/mL, resulting in an approximate class imbalance ratio of 4:1. This imbalance highlights the skewed distribution of the data, which required careful consideration during model development to ensure reliable predictions.

**Figure 1:**
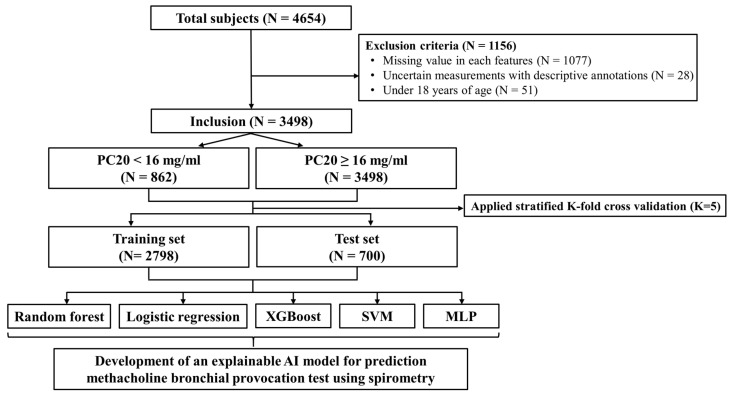
Flowchart of the data preprocessing.

Additionally, to predict the provocative concentration causing a 20% fall in FEV_1_ (PC20) results using only various spirometry outcomes, we excluded patient demographics such as sex, age, height, and weight from the analysis. Data with missing MBPT results or incomplete values for the variables were also excluded, as shown in Figure 1. To ensure consistency in the input features, all variables were normalized to a range of [−1, 1] using a Min–Max Scaler, which was implemented using Python version 3.8.10.

### 2.3. Prediction Models

We employed five machine learning models to predict MBPT outcomes based on pulmonary function test data. The performance of these models was evaluated using 5-fold Stratified Cross-Validation [26], a robust method that divides the dataset into k equally sized folds. For each iteration, one fold was designated as the validation set, while the remaining k − 1 folds were used for training. This process was repeated k times, ensuring that each data point was used exactly once for validation and k − 1 times for training.

Performance metrics, including AUC, accuracy, F1-score, precision, and recall, were calculated for each fold. We reported the mean values of these metrics along with 95% confidence intervals, providing a comprehensive evaluation of the model’s generalization capability. This approach ensures a reliable assessment by leveraging the entire dataset during cross-validation.

In our implementation, we divided the dataset into five folds, carefully maintaining the class distribution across the folds using stratified sampling. During each fold, the dataset was split into training (80%) and validation (20%) subsets, preserving the original class distribution to mitigate potential biases arising from imbalanced data. This iterative process allowed for comprehensive utilization of the dataset, minimizing the risk of overfitting or underestimating model performance.

RF [27] is an ensemble learning method that constructs multiple decision trees during training, and aggregates their outputs to make predictions. By employing techniques such as bootstrapping and random feature selection for each tree, RF is robust against noise and feature collinearity, reducing the risk of overfitting. Additionally, RF is versatile, handling both classification and regression tasks, and it provides insights into feature importance through its hierarchical structure.

LR [28] is a simple yet effective linear model designed for binary classification. It computes the probability of an outcome y using the logistic function

$$P\left(y=1|X\right)=\frac{1}{1+e^{-\left(\beta_{0}+\beta_{1}X_{1}+\beta_{1}X_{1}+\cdots +\beta_{n}X_{n}\right)}}$$

where *X*_1_, *X*_2_, …, *X*_*n*_ are the features, *β*_0_ is the intercept, and *β*_1_, *β*_2_, …, *βn* represent the contribution of each feature to the outcome.

Owing to its interpretability and fast training speed, LR is particularly useful for datasets where linear separability is reasonable. It is simple and computationally efficient; however, LR may underperform on non-linear or complex datasets. Enhancements such as incorporating quadratic terms ($X^{2}$) to model non-linear relationships between features and the outcome, interaction terms ($X_{1}\times X_{2}$) to capture the combined effect of two or more features, or step functions to segment the data into discrete intervals for more granular modeling, can improve its ability to model non-linear relationships.

XGBoost [29] is a gradient-boosting algorithm that constructs decision trees sequentially, with each tree correcting the errors of its predecessor. Its advanced techniques, including regularization, parallelized tree construction, and efficient handling of missing values to achieve high predictive performance, make it particularly effective for structured and large-scale datasets. However, careful hyperparameter tuning is essential to avoid overfitting.

An SVM [30] constructs hyperplanes in a high-dimensional space to effectively separate classes. Through kernel functions such as linear, polynomial, or radial basis functions, an SVM can model non-linear relationships in the data. This model is particularly advantageous for smaller datasets, where overfitting can be controlled through careful selection of kernel and regularization parameters.

An MLP [31] is a neural network that excels at capturing complex, non-linear patterns. Our MLP architecture included 20 input features, a single hidden layer with 32 neurons, and an output layer for binary classification. The ReLU activation function introduced non-linearity in the hidden layer, while a sigmoid activation function in the output layer computed probabilities.

The training utilized a binary cross-entropy loss function, with class imbalance addressed using weights calculated via the compute_class_weight function [32] from Scikit-learn [33] (version 1.3.2). An Adam optimizer with a learning rate of 0.002 was employed to adaptively adjust the learning rate. Early stopping, triggered after 20 epochs of no improvement in validation loss, prevented overfitting.

Additionally, we used 5-fold Stratified Cross-Validation to handle class imbalance and ensure that each fold preserved the distribution of positive and negative cases. This approach ensured robust evaluation, particularly given the class imbalance in our dataset. By incorporating these diverse models, we aimed to compare their performance comprehensively and identify the most suitable approach for predicting MBPT outcomes.

### 2.4. Evaluation

Model performance was evaluated using consistent metrics, including the F1-score, AUC, precision, recall, and accuracy, to provide a more comprehensive assessment of model performance.

The AUC, a key metric for binary classification, represents the area under the receiver operating characteristic (ROC) curve, where the *x*-axis denotes the false positive rate (FPR) and the *y*-axis the true positive rate (TPR). These rates are calculated as follows:

$$\mathrm{FPR}=\frac{\mathrm{False}\;\mathrm{Positives}\left(\mathrm{FP}\right)}{\mathrm{False}\;\mathrm{Positives}\left(\mathrm{FP}\right)+\mathrm{True}\;\mathrm{Negatives}\left(\mathrm{TN}\right)}$$

$$\mathrm{TPR}=\frac{\mathrm{True}\;\mathrm{Positives}\left(\mathrm{TP}\right)}{\mathrm{True}\;\mathrm{Positives}\left(\mathrm{TP}\right)+\mathrm{False}\;\mathrm{Negatives}\left(\mathrm{FN}\right)}$$

The F1-score is the harmonic mean of precision and recall, balancing their trade-offs. It is particularly valuable in scenarios where class imbalances exist, as it considers both false positives and false negatives.

$$F1-\mathrm{Score}=2\times \frac{\mathrm{precision}\times \mathrm{recall}}{\mathrm{precision}+\mathrm{recall}}$$

Precision indicates the proportion of true positive predictions out of all positive predictions made by the model.

$$\mathrm{precision}=\frac{\mathrm{True}\;\mathrm{Positives}\left(\mathrm{TP}\right)}{\mathrm{True}\;\mathrm{Positives}\left(\mathrm{TP}\right)+\mathrm{False}\;\mathrm{Positives}\left(\mathrm{FP}\right)}$$

Recall, equivalent to TPR and also referred to as sensitivity, is the proportion of actual positive cases correctly identified by the model.

Accuracy represents the proportion of correct predictions (both true positives and true negatives) among all predictions.

$$\mathrm{accuracy}=\frac{\mathrm{True}\;\mathrm{Positives}\left(\mathrm{TP}\right)+\mathrm{True}\;\mathrm{Negatives}\left(\mathrm{TN}\right)}{\mathrm{Total}\;\mathrm{Predictions}\left(\mathrm{TP}+\mathrm{TN}+\mathrm{FP}+\mathrm{FN}\right)}$$

### 2.5. Statistical Analysis

Differences in distribution between the PC20 < 16 mg/mL and PC20 ≥ 16 mg/mL groups were confirmed using the Mann–Whitney U test. Results are presented as median (Q1, Q3).

## 3. Results

### 3.1. Baseline Characteristics

A total of 862 patients in the PC20 < 16 mg/mL group and 2636 patients in the PC20 ≥ 16 mg/mL group were included in the study. As shown in Table 1, statistically significant differences were observed between the two groups for various pulmonary function measures, including pre- and post-FEV_1_, and FEF_25–75_ (all *p*-values < 0.001).

The PC20 < 16 mg/mL group exhibited decreased lung function across most indicators, with a particularly pronounced reduction in small airway flow measures such as FEF_25–75_. By contrast, the negative control group demonstrated higher lung function values.

### 3.2. Predictive Performances

**Figure 2:**
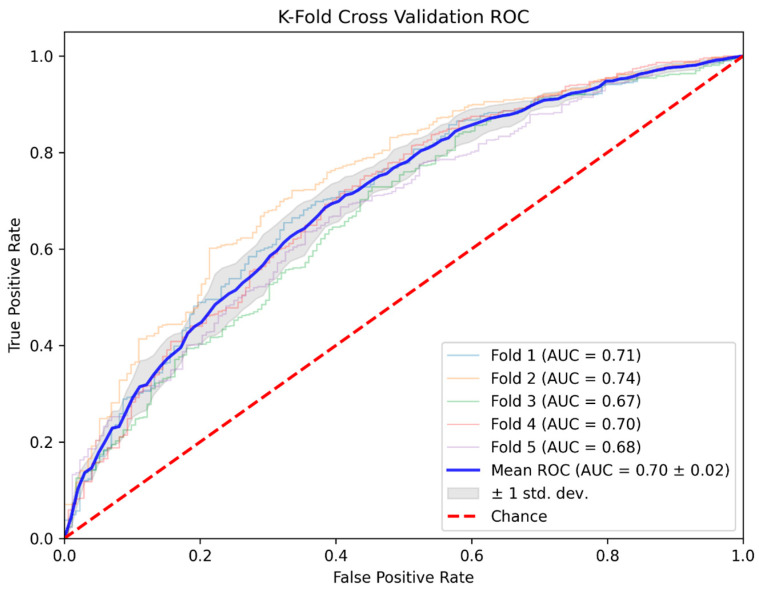
Receiver operating characteristic curve of MLP model with stratified K-fold (K = 5) cross validation.

Figure 2 shows the average ROC curve for the MLP model, with a mean AUC of 0.701 (95% CI: 0.676–0.725). Performance comparisons of all models are summarized in Table 2. The performance of each model was evaluated based on key metrics, including the AUC, accuracy, F1-score, precision, and recall.

As shown in Table 2 and Figure 2, the MLP achieved an AUC of 0.701, indicating acceptable discrimination for distinguishing between cases with PC20 < 16 mg/mL and PC20 ≥ 16 mg/mL. This performance highlights the model’s ability to effectively identify the minority class (PC20 < 16 mg/mL) and reduce false negatives, which is critical when dealing with class-imbalance datasets. The LR and SVM both achieved AUC values of 0.688, demonstrating comparable performance levels. Meanwhile, RF and XGBoost achieved slightly lower AUCs of 0.669 and 0.672, respectively, though both models still performed satisfactorily. These findings underscore the MLP model’s relative advantage in handling imbalanced data and improving sensitivity to rare cases.

**Figure 3:**
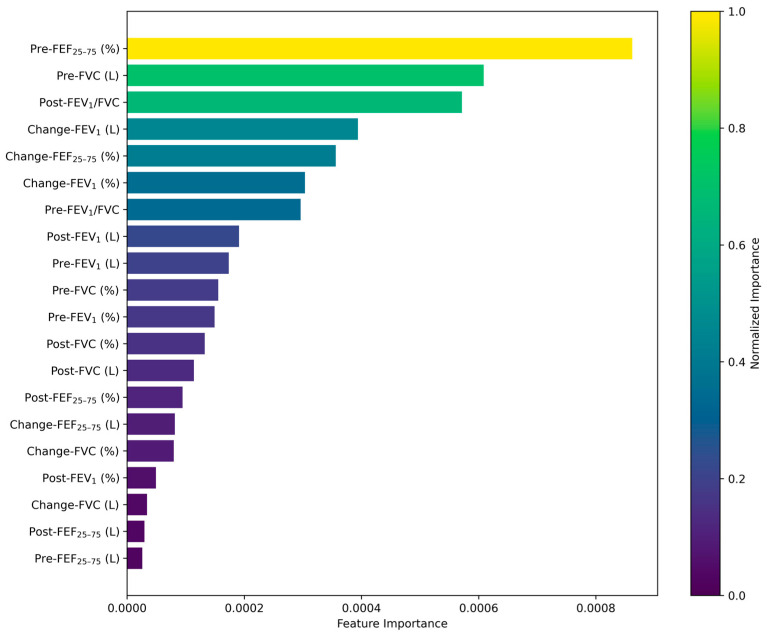
Feature importance of the input variables in the MLP model.

Figure 3 illustrates the key features identified by the MLP model as significant contributors to predicting PC20 test results. The most important features identified by the MLP model were Pre-FEF_25–75_ (%), Pre-FVC (L), Post-FEV_1_/FVC, Change-FEV_1_ (L), and Change-FEF_25–75_ (%). These variables aligned with the pulmonary function measures that showed significant differences between the asthma and non-asthma groups in the baseline analysis.

**Figure 4:**
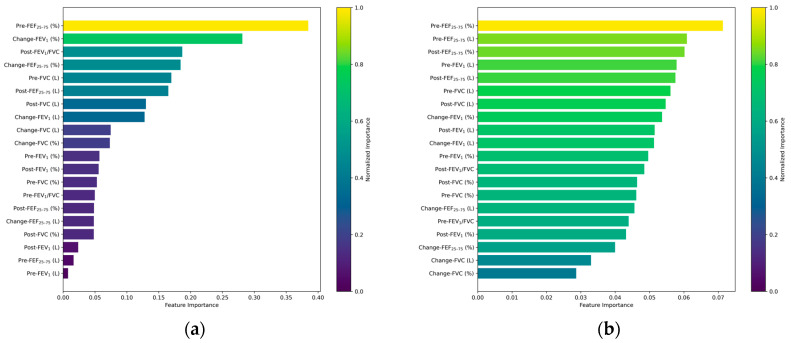

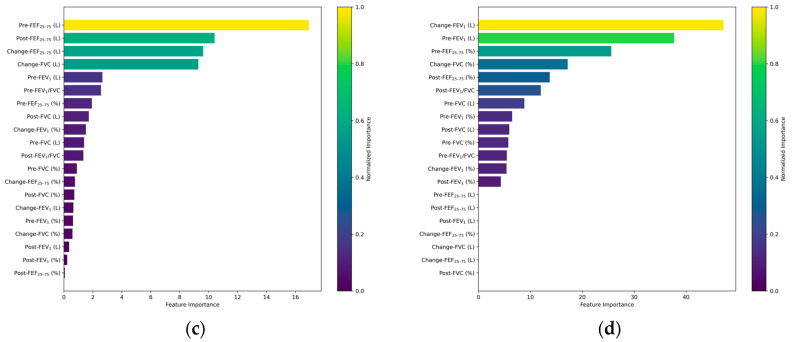
Feature importance of the input variables in the various other models: (**a**) logistic regression, (**b**) random forest, (**c**) SVM, and (**d**) XGBoost.

Figure 4 highlights the feature importance of the LR, RF, SVM, and XGBoost models, identifying the top five variables for each model. For the LR model, the top five variables were Pre-FEF_25–75_ (%), Change-FEV_1_ (%), Post-FEV_1_/FVC, Change-FEF_25–75_ (%), and Pre-FVC (L) These results align with the baseline characteristics, which showed significant differences in these variables between the PC20 < 16 mg/mL and PC20 ≥ 16 mg/mL groups (all *p*-values < 0.001). The LR model, as a linear model, is particularly sensitive to features with pronounced statistical differences between groups. Variables such as Pre-FEF_25–75_ (%) and Change-FEV_1_ (%), which exhibited pronounced variability in baseline measures, are strong predictors due to their consistent patterns in distinguishing predicting PC20 results.

For the RF model, the top five variables were Pre-FEF_25–75_ (%), Pre-FEF_25–75_ (L), Post-FEF_25–75_ (%), Pre-FEV_1_ (L), and Post-FEF_25–75_ (L). Unlike the LR model, the RF model leverages the power of ensemble learning to capture complex, non-linear relationships within the data. The dominance of FEF_25–75_-related variables reflects their sensitivity in identifying subtle differences in airway function, as indicated in the baseline analysis. The RF model’s reliance on multiple decision trees appears to balance the contribution of each variable, leading to relatively uniform importance values. This pattern suggests that RF may be better suited for datasets with high inter-variable correlation, as seen in spirometry measurements.

The SVM model’s top variables were Pre-FEF_25–75_ (L), Post-FEF_25–75_ (L), Change-FEF_25–75_ (L), Change-FVC (L), and Pre-FEV_1_ (L). The SVM, being a kernel-based model, emphasized boundary definitions in high-dimensional spaces. The prominence of FEF_25–75_ (L) and FVC-related features reinforces their sensitivity in identifying subtle differences in pulmonary function that are critical for MBPT prediction. The statistical significance, as presented in Table 1, underscores their utility in differentiating between PC20 < 16 mg/mL and PC20 ≥ 16 mg/mL cases.

For the XGBoost model, the top variables were Change-FEV_1_ (L), Pre-FEV_1_ (L), Pre-FEF_25–75_ (%), Change-FVC (%), and Post-FEF_25–75_ (%). The XGBoost model’s sequential learning approach amplifies the influence of features that correct prior prediction errors, leading to higher importance for variables like Change-FEV_1_ (L) and Pre-FEV_1_ (L). These variables showed significant baseline differences (*p* < 0.001) and play a pivotal role in capturing dynamic changes in airway function, which are crucial for MBPT outcome prediction.

Across the models, Pre-FEF_25–75_ (L) and Pre-FEF_25–75_ (%) consistently ranked among the most influential features, except for the XGBoost model. These variables are clinically and statistically significant markers of small airway obstruction and exhibited markedly lower values in the PC20 < 16 mg/mL group (median (Q1, Q3): 2.19 (1.44, 2.98) L) compared to the PC20 ≥ 16 mg/mL group (2.64 (1.86, 3.53) L).

Tree-based models like RF and XGBoost demonstrated distinct patterns of feature importance. While RF exhibited balanced contributions from multiple variables due to its averaging approach, XGBoost concentrated its importance on a few key features. For instance, Change-FEV_1_ (L) emerged as the most critical variable, likely due to its strong correlation with dynamic airway changes that are essential for MBPT prediction. The difference in how these tree-based models treat feature importance highlights their distinct mechanisms for learning from data.

The findings from the feature importance analysis resonate with the baseline characteristics of the study population. Variables like FEF_25–75_, FEV_1_, and FVC consistently showed significant statistical differences between the groups, reinforcing their predictive value across models. This strong alignment between baseline characteristics and model-driven feature importance underscores the robustness of the selected input features and their clinical relevance.

Moreover, the influence of features like Pre-FEF_25–75_ and Change-FEV_1_ across multiple models reflects their ability to capture both static and dynamic aspects of airway functioning, which are critical for accurately predicting MBPT outcomes. These variables’ consistent impact across diverse algorithms highlights their potential as universal markers for asthma diagnosis in AI-driven clinical applications.

## 4. Discussion

This study aimed to develop an AI model to predict MBPT results using FEV_1_ and bronchodilator response (BDR) measurements from spirometry. Key variables used in the model included PC20 (the target outcome), along with various spirometry and BDR test results. These variables included pre- and post-bronchodilator FVC (L and %), FEV_1_ (L and %), FEF_25–75_ (L and %), and FEV_1_/FVC (L and %), as well as their respective percent and absolute changes. The AI model demonstrated acceptable performance, with an AUC of 0.701 (95% CI: 0.676–0.725), F1-score of 0.853, and accuracy of 0.758.

Feature importance analysis of the MLP model highlighted the clinical relevance of variables such as Pre-FEF_25–75_ (%), Pre-FVC (L), Post-FEV_1_/FVC, percent change in FEV_1_, and percent change in FEF_25–75_. These findings highlight the potential of the model to effectively utilize spirometry data, particularly FEV_1_ and BDRs, for accurate MBPT predictions. By minimizing reliance on MBPT, this AI-based approach could streamline asthma diagnostic workflows, facilitating faster and more accessible assessments.

Conventional asthma diagnosis involves a combination of tests, including spirometry, asthma provocation tests, and sputum analysis, along with clinical symptomatology [34]. When spirometry with BDR testing yields inconclusive results, particularly in routine cases, MBPT is often employed to assess airway hyper-responsiveness and confirm an asthma diagnosis [35]. However, MBPT has limitations [36], including its unsuitability for patients with severe airway obstruction (FEV_1_ < 50% predicted or <1.0 L), recent cardiovascular events (within the past 3 months), uncontrolled hypertension, and aortic aneurysms. Additional minor contraindications include moderate airway obstruction (FEV_1_ < 60% predicted or <1.5 L), inability to perform spirometry of acceptable quality, pregnancy, or breastfeeding, and the use of cholinesterase inhibitors for conditions such as myasthenia gravis [37]. Practical challenges, such as the need for specialized facilities and the discontinuation of medications or foods that may influence bronchial responsiveness along with proper storage and preparation of methacholine, further complicate MBPT implementation, particularly in resource-limited settings [38]. To address these challenges, we developed an AI model capable of predicting MBPT results using spirometry and BDR data. This model offers an alternative to MBPT, potentially enhancing clinical decision-making and improving asthma diagnostics in settings where MBPT is less accessible.

Recent advancements in AI technology have revolutionized medical data analysis across various fields [39]. Unlike traditional statistical methods, AI algorithms excel in providing higher accuracy, handling large-scale datasets, and uncovering intricate non-linear relationships [39]. In respiratory medicine, AI has demonstrated considerable potential, particularly in the evaluation of imaging data for lung cancer, diagnosis of fibrotic lung diseases, and analysis of subtle imaging changes that might elude human interpretation [40]. Moreover, AI has been increasingly applied to pulmonary function tests, aiding in the precise diagnoses of obstructive and restrictive lung diseases such as chronic obstructive pulmonary disease (COPD), asthma, and idiopathic pulmonary fibrosis. Studies have shown that AI algorithms play a pivotal role in improving the prediction and diagnosis of respiratory diseases, offering tools to classify asthma phenotypes and severity, predict acute exacerbations, and optimize the performance of diagnostic methods. These advancements have not only streamlined diagnostic workflows, but also hold promise for enhancing personalized treatment strategies and improving patient outcomes. Building on this progress, our study contributes to the field by developing an AI model to predict MBPT results based on pulmonary function test data. We anticipate that this model could provide clinicians with a more efficient and accurate tool for assessing airway hyperresponsiveness, thereby supporting earlier interventions and better management of respiratory conditions.

Among the five tested models, the MLP model outperformed others in terms of AUC, accuracy, and precision. The use of the compute_class_weight function effectively addressed class imbalance, enhancing the model’s robustness.

While the MLP model is particularly well suited for clinical applications, where precision is critical to avoid false positives, its ability to minimize the misclassification of negative cases makes it highly valuable for screening purposes. However, despite its strengths, class imbalance (a 1:4 ratio of PC20 < 16 mg/mL to PC20 ≥ 16 mg/mL cases) remains a challenge. Future research should focus on addressing this issue through resampling techniques, adjusted class weights, or other strategies to optimize performance. Reducing class imbalances would enhance the model’s reliability, particularly in clinical settings where misclassification can have remarkable consequences. We aimed to provide further support for clinical decision-making and to improve patient outcomes by creating a model that can directly differentiate between PC20 < 16 mg/mL and PC20 ≥ 16 mg/mL cases. This approach would assist clinicians in identifying and managing asthma more accurately by comprehensively addressing both screening and diagnosis. In addition, the feature importance analysis further supports the interpretability and clinical applicability of the MLP model by identifying variables directly linked to airway hyperresponsiveness. Key variables such as Pre-FEF_25–75_ (%), Pre-FVC (L), Post-FEV_1_/FVC, percent change in FEV_1_, and percent change in FEF_25–75_ align with core diagnostic measures used in asthma evaluation. These insights reinforce the model’s potential to assist clinicians in making more accurate and efficient diagnostic decisions.

A key strength of our study is its multicenter design, which involved collaboration among medical institutions across Korea. This approach provided a diverse and representative dataset, encompassing various clinical practices, patient demographics, and healthcare environments. Incorporating external validation further strengthens the AI model by ensuring its generalizability across different clinical settings, addressing a limitation often encountered in previous research.

## 5. Conclusions

This study successfully developed an AI model to predict MBPT results using spirometry data combined with BDR measurements.

AI-based models, like the one developed in our study, have the potential to reduce reliance on MBPT by offering a faster and more accessible method to evaluate airway hyperreactivity, particularly in resource-limited settings. The ability of this model to leverage readily available spirometry data provides a promising alternative for enhancing the efficiency and accuracy of asthma diagnosis.

However, further refinements are required to address class imbalances and improve the model’s performance in broader clinical applications. Future studies should focus on resolving these issues and improving the model’s interpretability to realize its potential in clinical practice fully.

This AI model represents a valuable tool for asthma diagnosis, enabling clinicians to consider the likelihood of asthma by predicting PC20 < 16 using spirometry with BDR alone, without the need for MBPT testing. By facilitating more convenient and efficient patient diagnosis and treatment, the model has the potential to enhance clinical workflows and improve patient care outcomes.

## Figures and Tables

**Table 1:** Baseline characteristics.

Variables (Median (Q1, Q3))	PC20 < 16 mg/mL (*n* = 862 (24.64%))	PC20 ≥ 16 mg/mL (*n* = 2636 (75.36%))	*p*-Value
Pre-FVC (L)	3.09 (2.44, 3.79)	3.26 (2.64, 4.08)	<0.001
Pre-FVC (%)	85.00 (76.00, 94.00)	87.00 (79.00, 96.00)	<0.001
Post-FVC (L)	3.12 (2.50, 3.88)	3.24 (2.64, 4.07)	<0.001
Post-FVC (%)	85.00 (78.00, 95.00)	87.00 (79.00, 95.25)	0.031
Change-FVC (%)	1.00 (0.00, 3.00)	0.00 (0.00, 2.00)	<0.001
Change-FVC (L)	0.02 (0.00, 0.09)	0.00 (0.00, 0.06)	<0.001
Pre-FEV_1_ (L)	2.45 (1.87, 3.05)	2.63 (2.11, 3.31)	<0.001
Pre-FEV_1_ (%)	84.00 (75.25, 92.00)	89.00 (81.00, 97.00)	<0.001
Post-FEV_1_ (L)	2.52 (1.95, 3.19)	2.67 (2.16, 3.39)	<0.001
Post-FEV_1_ (%)	87.00 (78.00, 96.00)	91.00 (82.00, 99.00)	<0.001
Change-FEV_1_ (%)	4.00 (1.00, 7.00)	2.00 (0.00, 4.00)	<0.001
Change-FEV_1_ (L)	0.09 (0.03, 0.17)	0.05 (0.00, 0.11)	<0.001
Pre-FEF_25–75_ (L)	2.19 (1.44, 2.98)	2.64 (1.86, 3.53)	<0.001
Pre-FEF_25–75_ (%)	73.00 (57.00, 92.75)	89.00 (70.00, 108.00)	<0.001
Post-FEF_25–75_ (L)	2.53 (1.68, 3.45)	2.93 (2.08, 3.90)	<0.001
Post-FEF_25–75_ (%)	85.00 (67.00, 104.00)	99.00 (78.00, 118.00)	<0.001
Change-FEF_25–75_ (%)	14.00 (5.00, 25.00)	11.00 (3.00, 19.00)	<0.001
Change-FEF_25–75_ (L)	0.29 (0.10, 0.55)	0.26 (0.07, 0.49)	0.002
Pre-FEV_1_/FVC	78.00 (72.25, 84.00)	81.00 (76.00, 85.00)	<0.001
Post-FEV_1_/FVC	81.00 (75.00, 86.00)	83.00 (78.00, 87.00)	<0.001

FVC: forced vital capacity; FEV_1_: forced expiratory volume in one second, FEF_25–75_: forced expiratory flow between 25% and 75%; SD: standard deviation.

**Table 2:** Performance of various machine learning models.

	AUC (95% CI)	F1-Score	Accuracy	Precision	Recall
RF	0.669	0.854	0.753	0.771	0.957
	(0.635–0.703)	(0.847–0.861)	(0.766–0.775)	(0.742–0.763)	(0.943–0.971)
LR	0.688	0.861	0.761	0.765	0.986
	(0.654–0.721)	(0.858–0.864)	(0.755–0.767)	(0.759–0.771)	(0.978–0.993)
XGBoost	0.672	0.860	0.754	0.754	1.0
	(0.643–0.701)	(0.859–0.860)	(0.753–0.754)	(0.753–0.754)	(1.000–1.000)
SVM	0.688	0.860	0.755	0.757	0.995
	(0.653–0.723)	(0.859–0.861)	(0.752–0.758)	(0.754–0.760)	(0.991–0.999)
MLP	0.701	0.853	0.758	0.787	0.932
	(0.676–0.725)	(0.848–0.858)	(0.754–0.762)	(0.776–0.797)	(0.906–0.957)

## Data Availability

Data sharing was possible after obtaining approval from the Institutional Review Board and Data Review Committee of the Kangwon National University Hospital, Seoul Medical Center, Dongguk University Ilsan Hospital, and Hallym University Dong-tan Sacred Heart Hospital.

## Abbreviations

CVA	cough variant bronchial asthma
FEV_1_	forced expiratory volume in one second
SABA	short-acting b2-agonist
FEF_25–75_	forced mid-expiratory flow between 25% and 75%
FVC	forced vital capacity
MBPT	methacholine bronchial provocation test
AI	artificial intelligence
PC20	predict provocative concentration causing a 20% fall in FEV_1_
RF	random forest
LR	logistic regression
XGBoost	extreme gradient boosting
SVM	support vector machine
MLP	multi-layer perceptron
AUC	area under the curve
CI	confidence interval
